# Carbon Consumption Patterns of Microbial Communities Associated with *Peltigera* Lichens from a Chilean Temperate Forest

**DOI:** 10.3390/molecules23112746

**Published:** 2018-10-24

**Authors:** Katerin Almendras, Diego Leiva, Margarita Carú, Julieta Orlando

**Affiliations:** Laboratory of Microbial Ecology, Department of Ecological Sciences, Faculty of Sciences, Universidad de Chile, Santiago 7800003, Chile; katalmendras@gmail.com (K.A.); dleiva@ug.uchile.cl (D.L.); mcaru@uchile.cl (M.C.)

**Keywords:** BioLog^®^ EcoPlate, community level physiological profiles, lichen microbiota, lichen substrate, *Nothofagus* forest

## Abstract

Lichens are a symbiotic association between a fungus and a green alga or a cyanobacterium, or both. They can grow in practically any terrestrial environment and play crucial roles in ecosystems, such as assisting in soil formation and degrading soil organic matter. In their thalli, they can host a wide diversity of non-photoautotrophic microorganisms, including bacteria, which play important functions and are considered key components of the lichens. In this work, using the BioLog^®^ EcoPlate system, we studied the consumption kinetics of different carbon-sources by microbial communities associated with the thallus and the substrate of *Peltigera* lichens growing in a Chilean temperate rain forest dominated by *Nothofagus pumilio*. Based on the similarity of the consumption of 31 carbon-sources, three groups were formed. Among them, one group clustered the microbial metabolic profiles of almost all the substrates from one of the sampling sites, which exhibited the highest levels of consumption of the carbon-sources, and another group gathered the microbial metabolic profiles from the lichen thalli with the most abundant mycobiont haplotypes. These results suggest that the lichen thallus has a higher impact on the metabolism of its microbiome than on the microbial community of its substrate, with the latter being more diverse in terms of the metabolized sources and whose activity level is probably related to the availability of soil nutrients. However, although significant differences were detected in the microbial consumption of several carbon-sources when comparing the lichen thallus and the underlying substrate, d-mannitol, l-asparagine, and l-serine were intensively metabolized by both communities, suggesting that they share some microbial groups. Likewise, some communities showed high consumption of 2-hydroxybenzoic acid, d-galacturonic acid, and itaconic acid; these could serve as suitable sources of microorganisms as bioresources of novel bioactive compounds with biotechnological applications.

## 1. Introduction

Lichens have been classically defined as mutualistic symbiotic associations where a fungus (mycobiont) provides a suitable habitat for an extracellular photobiont, either a green alga (chlorobiont) or a cyanobacterium (cyanobiont), or both. The photobiont fixes carbon through photosynthesis and, in the case of a cyanobacterium, also contributes to the fixation of nitrogen [1]. Approximately one-fifth of all known fungi have been described as obligate lichen-forming species [2], reflecting the evolutionary success of this symbiotic association. Lichens exist as discrete thalli characterized by a poikilohydric lifestyle, allowing them to grow in virtually any terrestrial environment, from the tropics to polar regions, covering up to 8% of the total land surface [1,3,4], with a few also occurring in freshwater or even submerged in marine environments [5,6]. Lichens play important roles in soil formation through weathering of rocks, leaching of minerals, stabilization of substrate particles formed by erosion, and helping to retain soil water [7]. In addition, they participate in the retention and distribution of nutrients (e.g., carbon, nitrogen) and trace elements [8,9].

One of the best-studied lichen genera is *Peltigera*. Lichens from this genus have been the subject of many recent studies in aspects such as biogeography [10], ecology [11,12,13], and specificity of symbionts [14,15], among others. Most *Peltigera* species are bipartite symbioses between the lichen-forming fungus and a cyanobacterium that belongs to the genus *Nostoc*; however, a few species are tripartite symbioses involving a green alga of the genus *Coccomyxa* as the main photobiont and *Nostoc* as the secondary photobiont growing in cephalodia [16,17]. These lichens commonly occur in humid, mainly shaded habitats, growing on soil, on peat, or among mosses [16,18]. Although these foliose lichens are readily recognized in the field through the identification of distinctive morphological characteristics, the genus is a taxonomically complex group and many challenges remain at the species level [10,17,19,20]. Currently, eight monophyletic sections within the genus *Peltigera* are recognized: *Chloropeltigera*, *Hydrothyriae*, *Horizontales*, *Peltidea*, *Peltigera*, *Phlebia*, *Polydactylon*, and *Retifoveatae* [17]. Recently, comprehensive phylogenetic revisions of sections *Polydactylon* [14], *Peltigera* and *Retifoveatae* [10] have been performed using sequence data from hundreds of specimens around the world. These analyses considerably increased the number of known species in these three groups, with most newly-delimited species restricted to a single biogeographical region and a few species with a near-cosmopolitan distribution. In Chile, only bipartite *Peltigera* cyanolichens of sections *Horizontales*, *Peltigera*, and *Polydactylon* have been reported, based on morphological characterizations [21,22] and phylogenetic analyses [10,14,23,24].

Lichens present a variety of growth forms, with external and internal surfaces where secondary metabolites are released, thus providing a great diversity of micro-niches for other eukaryotic and prokaryotic microorganisms, such as fungi and non-photoautotrophic bacteria [25,26,27]. Bacterial communities colonize lichens in a biofilm-like manner and are host specific, suggesting that lichen-associated bacteria are an integral component of lichen thalli. In fact, it has been suggested that the classical view of a dual symbiosis should alter, and thus consider the lichen symbiosis as multispecies interactions [25,28]. Metagenomic and proteomic studies have been used to propose the possible functions of the lichen microbiome; these include resistance against pathogens, stimulation of growth, degradation of older parts of the lichen thalli, and acquisition of nutrients (e.g., nitrogen, phosphorus, sulfur, and metals), among others [29,30,31,32].

One approach to characterize the metabolic activity of lichen-associated bacteria is to analyze the pattern of carbon-source utilization using the BIOLOG EcoPlate™ system. In this system, direct inoculation of community samples into wells with a variety of known carbon compounds allows the measurement of potential community carbon-source utilization, since the metabolism of the carbon-source is coupled to the capture of electrons by colorless tetrazolium salts, forming reduced purple formazans which can be readily monitored [33]. New technologies for the analysis of the genetic diversity of microbial communities are constantly emerging; however, metabolic profiles are extensively used to assess the impact of biotic or abiotic factors on the functional diversity of microbial communities [34,35], since differences in genetic diversity are not necessarily reflected in changes in metabolic profiles [36,37] probably because only a few taxa are essential for ecosystem functioning, many are functionally redundant and most organisms are metabolically inactive [38].

Several factors have been identified as descriptors of the lichen bacterial microbiome structure, including both intrinsic and extrinsic factors [28,39,40,41]. In a previous study, we discovered that the metabolic structure of the bacterial microbiota in *Peltigera* thallus was influenced by mycobiont identity and by the production of phenoloxidase activity, while the metabolic structure of the bacterial microbiota present in the substrates where lichens grow was shaped by cyanobiont identity and the sampling site [42]. Here, we hypothesize that the lichen influences the carbon-source consumption pattern of the community associated with its thallus, but has less impact on the microbial community of its underlying substrate. For this, we analyzed the pattern of consumption of different carbon-sources as a measure of metabolic activity of microbial communities associated with both thallus and substrate of *Peltigera* lichens, in order to identify the mainly used carbon-sources which contribute to define the metabolic ability of each community and the differences between them.

## 2. Results and Discussion

Previously, 50 lichen samples from two forested sites in the Coyhaique National Reserve in southern Chile, were identified by phylogenetically related sequences of cyanobacterial small sub-unit (SSU) rDNA and fungal large sub-unit (LSU) rDNA with sequences previously reported for *Nostoc* and *Peltigera*. This way, we defined five cyanobiont (C01, C03, C10, C12, and C14) and six mycobiont (M1, M2, M4, M5, M6, and M8) haplotypes [42]. In our previous phylogenetic analysis [24], most of our mycobionts formed defined and well-supported monophyletic groups with some of the *Peltigera* species downloaded from the database: M1 was closely-related to *P. ponojensis*, M2 to *P. extenuata*, M4 to *P. rufescens*, and M6 to *P. frigida*. However, some of our sequences were closely related to more than one species, forming defined and well-supported monophyletic lineages. Thus, we used the name of the most emblematic species in the group to name the lineage: M5 was part of the *P. canina* lineage, and M8 of the *P. hymenina* lineage. According to updated analyses of the species of these lineages, M5 and M8 most-likely correspond to *P. ‘fuscopraetextata’* and *P. truculenta*, respectively [10,14].

**Figure 1:**
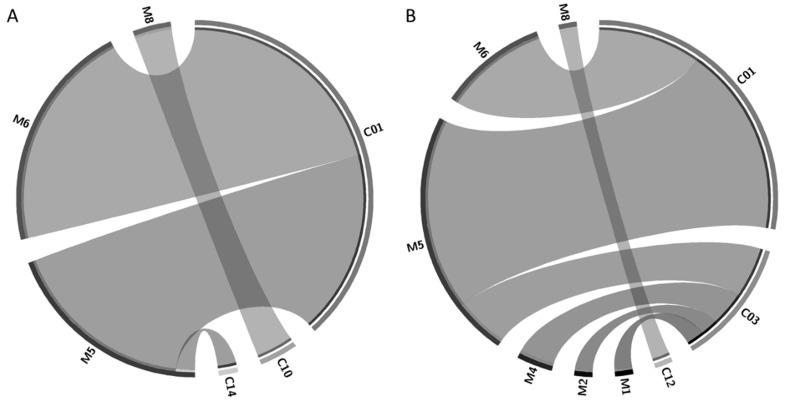
Circos graphic representations of symbiotic combinations of mycobiont (M) and cyanobiont (C) haplotypes in Site 1 (**A**) and Site 2 (**B**). The width of the ribbons represents the relative abundance of the symbiotic combinations that are connected. For reference, C14 and C12 have an abundance of one in Site 1 and Site 2, respectively.

Of these, three mycobiont (M5, M6, and M8) and one cyanobiont (C01) haplotypes were present in both sites. Regarding the symbiotic combinations (i.e., the analysis of which mycobiont is associated with which cyanobiont), four pairs were collected from site S1 (M5C01, M5C14, M6C01, and M8C10) and seven pairs were obtained from site S2 (M1C03, M2C03, M4C03, M5C01, M5C03, M6C01, and M8C12) (Figure 1). Two of them (M5C01 and M6C01) were the most abundant ones in both sites (21 and 18 out of 50, respectively). *P. ‘fuscopraetextata’* (related to M5), a species informally introduced by Miadlikowska et al. [20] and further supported by other studies [10,43], has been reported in Canada, USA, Argentina, and Chile [10]. On the other hand, *P. frigida* (related to M6) is restricted to southern Chile and Argentina [10,21,22,24]. Therefore, it is expected that both species are highly abundant in the extreme south of South America (Holantarctic Kingdom according to Martínez et al. [21] or Neantarctic Region according to Magain et al. [10]).

Although our study is confined to a particular geographic area, it is possible to observe some specificity patterns in the mycobiont–cyanobiont associations. In general, mycobionts are more specialized than cyanobionts, whereas cyanobionts associate frequently with several *Peltigera* species [10,12,14,15]. This is the case of C03, which is associated with four mycobionts in a reduced area, although the abundance of its pairs is low, so probably the sampled forest is not the optimum environment for this cyanobacterium. Conversely, those lichens paired to the successful cyanobiont C01, such as M5 (most probably *P. ‘fuscopraetextata’*) and M6 (*P. frigida*), were the most abundant. *P. ‘fuscopraetextata’* has asexual propagules (phyllidia) allowing the vertical transmission of the photobiont and thus high levels of specificity, which is in accordance with the results of Magain et al. [10]. In addition, these authors reported that *P. frigida* showed intermediate levels of specialization; although we found that this mycobiont just paired with the cyanobiont C01, when we extend the analysis to other sites, we actually observe that its specificity is not as high (e.g., in the Magallanes region, this mycobiont is paired with cyanobionts C02 and C14 [24]). On the other hand, M8 (most probably *P. truculenta*), from the *Polydactylon* section, was associated with specific cyanobionts (C10 and C12, plus C11 if the broader sampling sites in Zúñiga et al. [24] are considered), which were not paired with any other mycobiont [24]. This was also observed across the class *Lecanoromycetes*, where specific monophyletic groups of lichens are specialized on specific groups of photobionts [44,45]. More specifically, *Peltigera* species from section *Polydactylon* also have a high specialization [12,15] at narrow phylogenetic or geographic scales. Nevertheless, it has been shown previously [14] that most South American *Peltigera* species from this section are more generalists than other species, which would be advantageous for colonizing new geographical areas or habitats.

To carry out the metabolic profiling, composite samples from the lichen thallus and the associated substrate were prepared, grouping them by site (S1 and S2), mycobiont type (M), and cyanobiont type (C). This arrangement produced 11 samples from lichen thalli and 11 samples from the underlying substrates [42]. The kinetics of the consumption of carbon-sources of all samples were adjusted to the modified Gompertz equation, determining that at 48 h all the communities were in the exponential phase of color development (Appendix A). Thus, the communities analyzed in this study grow faster than those evaluated in other studies, where the exponential phase was reached only at 72 h [34,46]. This difference should not be due to the temperature of the test since these were similar to those found in natural conditions (28 °C and 25 °C). Therefore, it is likely that this difference in the growth rate is due to natural differences at the sampling sites, since it has been shown that environmental conditions can shape the metabolic structures of lichen bacterial microbiomes and bacterial communities of soils [34,40,46,47].

**Figure 2:**
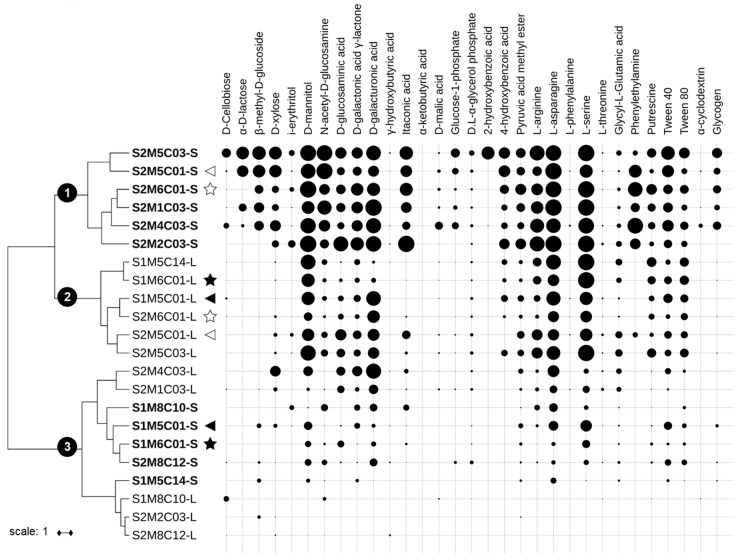
Ward’s clustering based on the metabolic profiles of the lichen and substrate microbiotas. Normalized carbon consumption data at the exponential phase and Euclidean distance were used. Consumption data by each sample is shown with a circle plot for each carbon-source, and the diameter of the circle is proportional to the magnitude of consumption. The nomenclature for each composite sample indicates the site (S1 or S2), then the mycobiont (M1, M2, M4, M5, M6, or M8), then the cyanobiont (C01, C03, C10, C12, or C14) and whether it comes from lichen thallus (-L) or from the substrate (-S). Substrate samples names are emphasized in bold. M5C01 samples are indicated with triangles and M6C01 with stars. Filled triangles and stars indicate M5C01 and M6C01 samples from Site 1, whilst empty triangles and stars indicate M5C01 and M6C01 samples from Site 2.

Subsequently, the consumption data of the 31 carbon-sources in EcoPlates^TM^ by the lichen and substrate microbiotas registered during the exponential phase (48–72 h) were used to carry out a clustering analysis to group bacterial communities with similar carbon-source utilization patterns (Figure 2). The results revealed that the communities were distributed in three groups (Groups 1–3).

Group 1 includes the microbial metabolic profiles of almost all the substrates from site S2 (except S2M8C12-S, which was clustered in Group 3). The consumption of carbon-sources indicates that significantly higher metabolic activity was detected in the substrate microbiotas, which utilized mainly the following sources: d-mannitol and *N*-acetyl-d-glucosamine (carbohydrates); d-galactonic acid γ-lactone, d-galacturonic acid, and itaconic acid (carboxylic acids); l-arginine, l-asparagine, and l-serine (amino acids); and phenylethylamine.

Group 2 gathered the microbial metabolic profiles associated with the lichen thallus with mycobiont haplotypes M5 and M6, the most abundant ones in both sites. The patterns grouped in this cluster were characterized by a generally lower consumption of some carbon-sources when compared to those from the substrates clustered in Group 1, but they used mainly d-mannitol and the amino acids l-asparagine and l-serine at a similar level.

Group 3 was the most heterogeneous one and included the metabolic profiles of five microbial communities obtained from lichen thalli (S2M4C03-L, S2M1C03-L, S1M8C10-L, S2M2C03-L, and S2M8C12-L) and five obtained from the substrates (S1M8C10-S, S1M5C01-S, S1M6C01-S, S2M8C12-S, and S1M5C14-S). This group exhibited the lowest levels of carbon-source consumption.

The carbon-source consumption pattern by the lichen substrate microbial communities from site S2 (Group 1) appears more functionally diverse and metabolically active compared to that of the community of the lichen thalli and substrates from site S1. This is in accordance with the features of site S2, which is closer than site S1 to wetlands, meaning that the communities obtained from substrates of site S2 were presumably exposed to higher levels of organic matter [42,48].

On the other hand, two of the lichen samples whose mycobiont haplotypes are present in both sites (M5 and M6) grouped in the same cluster (Group 2), which suggests that of the mycobiont and the site, the former has the greater influence on the microbial community of the thallus. Although soil factors certainly affect soil microbial communities [49], these factors could become less important in the case of soils influenced by lichens [50]. However, lichen samples with the mycobiont haplotype M8 (also present in both sites) did not group within this cluster, but clustered together with their substrates in Group 3. This mycobiont haplotype is the only one belonging to the section *Polydactylon*, whilst the other five are part of the section *Peltigera* [24]. Therefore, this metabolic differentiation could be a result of the distinct phylogenetic histories of each mycobiont section [17]. However, this metabolic differentiation could also be a consequence of the incidence of cyanobiont haplotypes C10 and C12, which show phylogenetic specificity with the mycobiont haplotype M8 [11]. The influence of photobionts on bacterial communities closely related to lichen thalli has been suggested before, as the photosynthetic and nitrogen-fixing capabilities of the photobionts could influence the availability of soil nutrients [40,42,50].

The carbon-sources that were intensively metabolized by both types of communities (i.e., from lichens and substrates) were d-mannitol, l-asparagine, and l-serine. Mannitol is the most abundant polyol in nature; it is produced by bacteria, yeasts, fungi, algae, lichens, and many plants, and is used as a carbon and energy source [51]. In addition, this carbohydrate is the most widely distributed polyol in fungi, being found in spores, fruiting bodies, and mycelia [52], which suggests that it could be very abundant in the soil of temperate rainy forests and therefore an important carbon-source for microbial communities from lichens and substrates. On the other hand, cyanobacteria and certain species of bacteria associated with lichens are able to liberate free amino acids [53]. Amino acids would become easily available to microbial communities during their growth and thus it is expected that a high utilization rate of amino acids is observed in these communities. Furthermore, considering that the metabolic abilities of microbial communities likely reflect the abundance and bioavailability of carbon compounds in the soil organic matter [54,55], the high use of amino acids suggests that they constitute an important energy source for soil microbial communities. Serine and asparagine are among the most abundant amino acids in the soil (5% of the total free amino acid content) [56]. On degradation to pyruvate and oxaloacetate, respectively, they become central in bacterial primary metabolism, and it is thus consistent that these carbon-sources were the most-consumed by both microbiotas.

Among the least consumed carbon-sources were 2-hydroxybenzoic acid, l-phenylalanine, and α-cyclodextrin. Aromatic compounds are stable in soils and are harder to degrade than many other organic compounds, since microorganisms require elaborate degradation strategies to overcome the high chemical stability of the aromatic ring [57]. However, it is interesting to highlight the high consumption of 2-hydroxybenzoic acid shown by the sample S2M5C03-S, since this acid is introduced into the environment because it is widely used as an intermediate in pharmaceuticals [58,59]. Since this aromatic organic compound is very toxic, further studies of its biodegradation by the soil microbial community underlying that lichen are needed to search for bacteria with potential applications in the bioremediation of environments contaminated with this pollutant.

On the other hand, some nutrients were more extensively utilized by the communities from the substrates than from the lichens, among them *N*-acetylglucosamine, d-galacturonic acid, itaconic acid, and phenylethylamine. These preferences of the communities of these Group 1 substrates could mean that these nutrients are available in the studied forest soils, in such a way that these microbial guilds have been enriched. *N*-acetylglucosamine is a building block of the bacterial peptidoglycan cell wall and is a monomeric unit in many naturally occurring polymers, such as chitin in the cell walls of many fungi and the exoskeleton or cuticle of arthropods; it is thus very abundant in most ecosystems. In addition, it plays an important role in supplying carbon and energy to bacteria by entering the glycolytic pathway after it is converted into fructose-6-phosphate [60,61]. d-galacturonic acid is one of the major polysaccharide constituents of plant cell walls, so it represents an important carbon-source for microorganisms living on decaying plant material, as found in the soil of temperate rain forests of southern Chile. In bacteria, this carboxylic acid is degraded in a five step pathway resulting in the formation of pyruvate and glyceraldehyde-3-phosphate [62]. Itaconic acid, an unsaturated dicarboxylic acid, and some alkylated derivatives are synthesized by some fungi and secreted in significant amounts to the environment [63]. Once secreted, this nutrient becomes available as a carbon and energy source by substrate-associated microbial communities. Finally, phenylethylamine is a microbial decarboxylation product of phenylalanine and can be found in fungi, bacteria [64], and many algae [65]. The physiological role of amine synthesis seems to be related to defence mechanisms used by bacteria to withstand acidic environments [66]. 

Two of the four abovementioned metabolites from Group 1 warrant particular attention, since it would be important to isolate microorganisms capable of metabolizing them in future work. First, we highlight d-galacturonic acid since it is the most abundant component of pectin, an abundant polysaccharide in plant cell walls. Pectin-rich residues, which are side products in sugar beet processing and in fruit juice production, are currently mainly used as animal feed, and it would be desirable to find new ways to convert this raw material into products of higher value [62]. Successful attempts have been described to ferment pectin-rich biomass to ethanol using genetically modified bacteria [67,68]. Therefore, exploring the microbial communities of specific lichen substrates could help find microorganisms that participate in such fermentations. Second, we draw attention to itaconic acid because it is of growing interest for the chemical industry as a renewable organic acid with potential to replace crude oil-based products (e.g., acrylic acid) [69]. While the anabolic pathway of this carboxylic acid is well understood, the catabolic pathway requires further research in order to engineer a production host with a disabled degradation pathway and thus increase its biodegradation potential [69].

**Figure 3:**
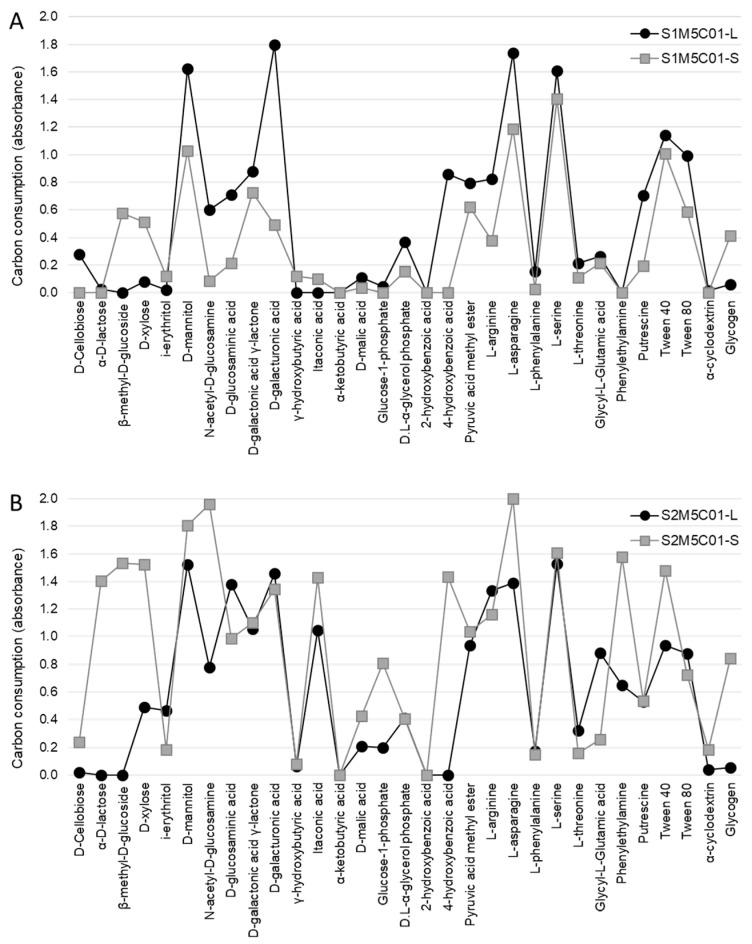
Metabolic profiles of M5C01. Consumption of the 31 carbon-sources by the lichen (-L) and substrate (-S) microbiotas associated with the symbiotic pair composed of the mycobiont haplotype M5 and the cyanobiont C01 haplotype from both sampling sites: S1 (**A**) and S2 (**B**).

**Figure 4:**
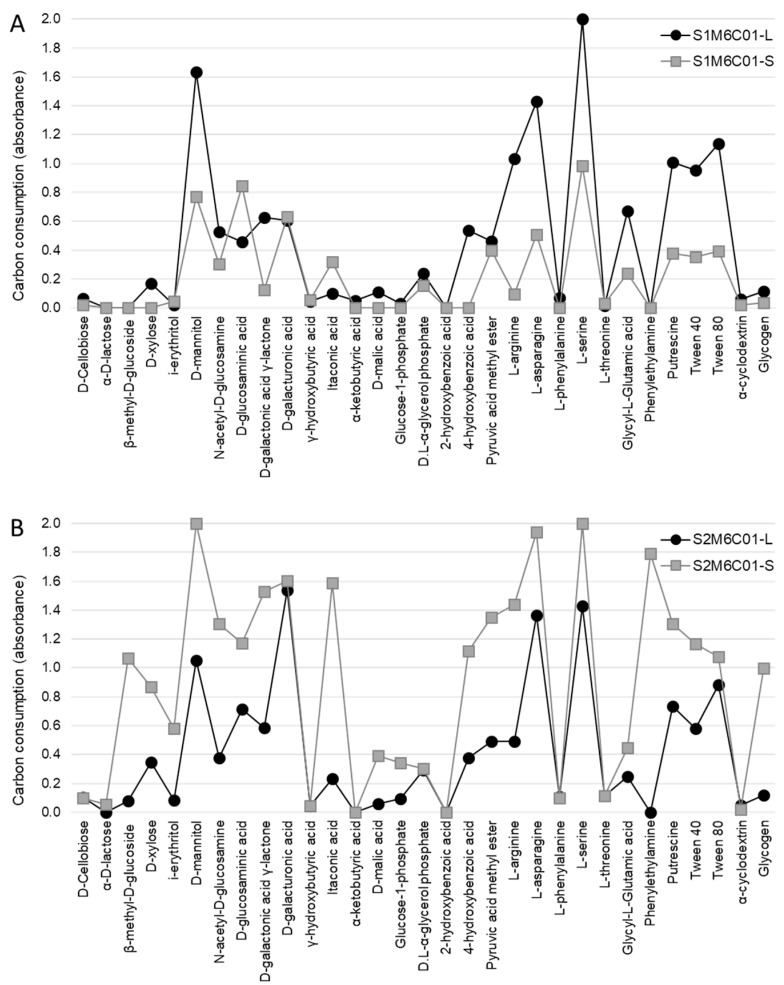
Metabolic profiles of M6C01. Consumption of the 31 carbon-sources by the lichen (-L) and substrate (-S) microbiotas associated with the symbiotic pair composed of the mycobiont haplotype M6 and the cyanobiont C01 haplotype from both sampling sites: S1 (**A**) and S2 (**B**).

Subsequently, we analysed samples M5C01 and M6C01 in more detail, since they were the most abundant symbiotic combinations at the two study sites, representing 95% and 68% of the samples collected in sites S1 and S2, respectively. Figure 3 shows the carbon-source consumption patterns of the microbial communities associated with M5C01 in site S1 (Figure 3A) and in site S2 (Figure 3B), which represent 43% and 44% of the samples collected at these sites, respectively. On the other hand, Figure 4 shows the carbon-source consumption patterns of the microbial communities associated with M6C01 in site S1 (Figure 4A) and in site S2 (Figure 4B), which represent 52% and 24% of the samples collected at these sites, respectively.

The consumption profiles show that both substrate communities exhibited higher activity at site S2 (Figure 3B and Figure 4B) compared with those at site S1 (Figure 3A and Figure 4A), which is consistent with the results previously reported by Leiva et al. [42], and suggests that edaphic factors have an effect on the level of metabolic activity of the soil microbial communities. On the other hand, when the microbial communities associated with the lichens are compared, the profiles are very similar depending on the mycobiont haplotype present in the thalli (M5, Figure 3; and M6, Figure 4) but independent of the site where the lichens grow. These results provide additional evidence that the lichen influences the metabolic pattern of the microbial community associated with its thallus, but has less impact on the microbial community of the underlying substrate.

Finally, when we statistically compared the consumption of each carbon-source by the microbiotas of these two lichen pairs (M5C01 and M6C01), we verified that 12 out of the 31 carbon-sources were consumed equally (d-Cellobiose, i-erythritol, d-mannitol, γ-hydroxybutyric acid, α-ketobutyric acid, d-malic acid, 2-hydroxybenzoic acid, l-phenylalanine, l-serine, l-threonine, Tween 80, and α-cyclodextrin) (Table 1). Few studies distinguish microbial communities associated with the thallus versus those associated with the substrates where lichens grow [41,42,70], but they all agree that bacteria associated with the lichen thalli could be recruited, at least in part, from the substrates where lichens grow.

In conclusion, the lichen thallus provides an adequate microhabitat that selects certain bacterial lineages [26], probably through the production of metabolites [42,71,72] which could select specific lineages in order to carry out defined functions as part of a multispecies symbiosis [25]. Several studies have characterized the biologically active metabolites produced by lichens (e.g., antibiotic, antiviral, anti-inflammatory, analgesic, cytotoxic, etc.) [71,73,74,75,76]; however, fewer studies isolated microorganisms associated with lichens as bioresources of novel bioactive compounds with biotechnological applications [77,78,79,80,81]. Here we propose that metabolic profiling could be used as a preliminary approach to select suitable samples to isolate microorganisms with specific metabolic features.

## 3. Materials and Methods

### 3.1. Study Sites and Sampling

Fifty *Peltigera*-thallus fragments (approximately 15 cm^2^ each) and their associated substrate (i.e., soil) (approximately 15 cm^3^ each) were collected from two plots 300 m away from each other (sites S1 and S2; approx. 2 Ha each) in a fragmented second-growth forest of *Nothofagus pumilio* [48] in the Coyhaique National Reserve (Aysén Region, Chile; 45°31′42.96′′ S, 72°1′51.95′′ W). These two sites are close to pine tree plantations, but site S2 is closer than site S1 to open spaces (i.e., rocky hillsides and a mallín, a kind of wetland) [42]. All samples were collected at least 1 m from the next closest thallus in order to minimize resampling of the same genetic individual. The samples were placed in paper bags and transported in cooled containers. In the laboratory, the lichen samples were stored in paper bags at room temperature, while the substrate samples (removed with a sterile brush and spatula) were sieved and stored in plastic tubes at 4 °C.

Lichen samples included in this study were previously identified by Zúñiga et al. [24]. The mycobiont and cyanobiont haplotypes were defined by analyzing the fungal LSU rDNA and cyanobacterial SSU rDNA regions, amplified with primers LIC24R and LR7 [17], and PCR1 and PCR18 [82], respectively. Phylogenetic analyses were performed with two sets of sequences; one set consisted of one representative of each mycobiont haplotype and 67 *Peltigera* sequences downloaded from GenBank, and the other set of sequences consisted of one representative of each cyanobacterial haplotype and 49 *Nostoc* sequences downloaded from GenBank. In addition, updated blast search analyses were performed in order to define the identity of mycobionts related to more than one reference species. Composite samples from both the lichen thallus and the associated substrate were prepared grouping the same mass of thallus or substrate of individual samples according to site (S1 and S2), mycobiont type (M) and cyanobiont type (C). Thus, the nomenclature for each composite sample indicates the site (S1 or S2), then the mycobiont (M1, M2, M4, M5, M6, or M8), then the cyanobiont (C01, C03, C10, C12, or C14) and whether it comes from the lichen thallus (-L) or from the substrate (-S).

### 3.2. Carbon-Source Utilization Pattern

The carbon-source utilization patterns of the microbial communities associated with lichen thalli and substrates were determined using EcoPlates (BioLog^®^, Hayward, CA, USA), containing 31 different carbon-sources (including 7 carbohydrates, 7 carboxylic acids, 2 phosphorylated chemicals, 2 aromatic compounds, 1 ester, 6 amino acids, 2 amines, and 4 polymers) plus one blank control, in triplicate. 

Microbial suspensions were obtained from 100 mg of thalli per composite sample, which were homogenized in 15 mL of PBS (137 mM NaCl, 2.7 mM KCl, 8 mM Na_2_HPO_4_, 2 mM KH_2_PO_4_) and shaken overnight at 150 rpm and 25 °C. In the same way, microbial suspensions were obtained from 2 g of substrates (i.e., soil) per composite sample, which were shaken at 150 rpm and 25 °C for 1 h in 20 mL of PBS. The plates were inoculated with 100 μL of the previously filtered microbial suspensions from thalli or substrates, and subsequently incubated at 25 °C for 1 week in a humid chamber. Carbon-source utilization was monitored every 24 h for 7 days at 590 nm in an Epoch microplate reader (Biotek, Winooski, VT, USA). Precision and uncertainty of the measurements of absorbance were calculated using a randomly chosen sample from each of both batches of samples (i.e., thalli and substrates) at each timepoint. Reproducibility (precision) of the analytical method was determined by estimating the relative standard deviation (RSD) of triplicate readings using the formula $\mathrm{RSD}=\left(s/\bar{x}\right)*100,$ where *s* is the standard deviation of the data set, and $\bar{x}$ is the average of the data set. In addition, the experimental uncertainty was estimated using the confidence interval (CI; 95% level of confidence) of triplicate readings using the formula $CI=\;\bar{x}\;\pm \left(ts/\surd n\right)$, where $\bar{x}$ is the average of the data set, *t* is the Student’s *t* for the desired level of confidence, *s* is the standard deviation of the data set, and *n* is the number of measurements (Table A1).

**Figure A1:**
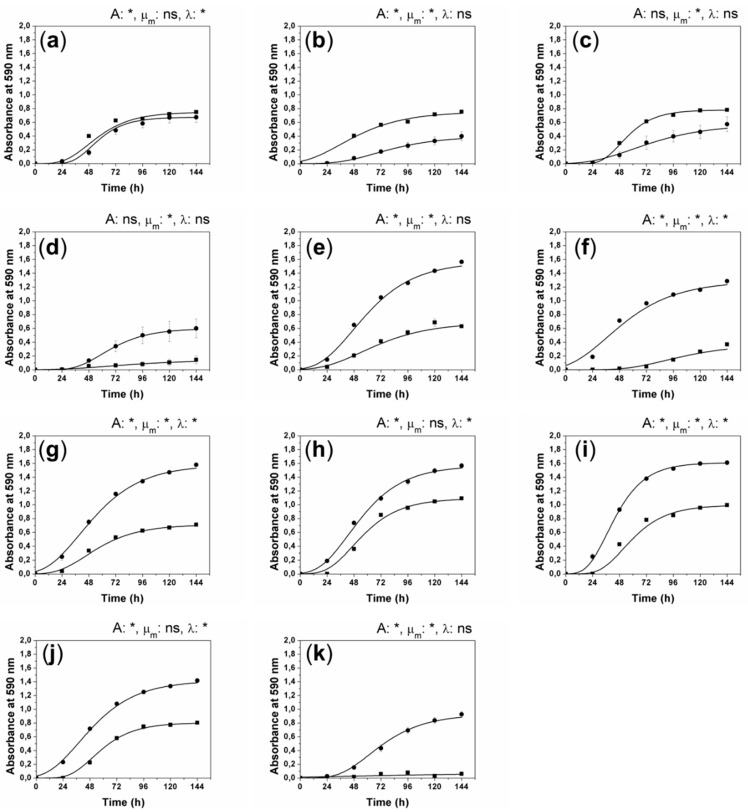
Colour development curves on the EcoPlate (Biolog) plates over time, adjusted according to the modified Gompertz model. λ, latency time (lag phase); μ_m_, maximum colour development rate; A, maximum absorbance. (**a**) S1M5C01, (**b**) S1M5C14, (**c**) S1M6C01, (**d**) S1M8C10, (**e**) S2M1C03, (**f**) S2M2C03, (**g**) S2M4C03, (**h**) S2M5C01, (**i**) S2M5C03, (**j**) S2M6C01, (**k**) S2M8C12. The nomenclature for each composite sample indicates the site (S1 or S2), then the mycobiont (M1, M2, M4, M5, M6, or M8), then the cyanobiont (C01, C03, C10, C12, or C14) and whether it originates from the lichen thallus(-L) or from the substrate (-S). The significantly-different values (*p* ≤ 0.05) for each of the parameters are indicated by an asterisk (*) when comparing the kinetics of the thalli (■) and the substrates (●).

Data were processed by subtracting the absorbance value at time zero, to minimize the interference of the sample colour [83], and the absorbance value from the control (water). In the data analysis, absorbance values of 0.1 or higher were considered positive and absorbance values higher than 2 were normalized to 2, according to the detection limit of the reader. To determine the incubation time at which all the communities were in the exponential phase of each curve and when the maximum absorbance was reached, data were adjusted using the modified Gompertz equation [84] using OriginPro software v8.07 (OriginLab Corporation, Northampton, MA, USA) (Figure A1). For the following analyses, the average of the data recorded at 48 h and 72 h for each carbon-source was used, since both plate reading times correspond to the exponential phase of the carbon-consumption kinetics.

### 3.3. Data Analyses

Circos plots representing the symbiotic combinations of mycobiont and cyanobiont haplotypes were obtained in Circos Table Viewer v0.63-9 [85].

Kinetic parameters (λ, μ_m_, and A) obtained from the modified Gompertz fitting were compared between each lichen and the corresponding substrate with one-way ANOVA comparisons with SPSS Statistics v17.0 (SPSS Inc, Chicago, IL, USA) since data showed a normal distribution according to the Jarque–Bera test and homoscedasticity of variances according to the Levene´s test.

Clustering of average carbon consumption data was performed in Past software v3.20 (University of Oslo, Oslo, Norway) [86], under Ward’s method with Euclidean distance. The resultant tree was exported, formatted in newick format and then imported in the iTOL v4.2.3 platform [87], where consumption data was added as a shape plot formatted dataset.

Since the data have a normal distribution according to the Jarque–Bera test (except those of α-d-Lactose consumption that had to be transformed by cubic root to have a normal distribution) but the assumption of homoscedasticity of variances was not fulfilled according to the Levene’s test, Welch’s *t*-test and Games–Howell post hoc test were used to evaluate the differences in the consumption of each carbon-source between the M5C01 and M6C01 samples, considering both lichen and substrate samples, using SPSS Statistics v17.0 (SPSS Inc, Chicago, IL, USA).

## Figures and Tables

**Table 1:** Statistical comparison of carbon consumption by lichen (-L) and substrate (-S) microbiotas of M5C01 and M6C01 from both sampling sites (S1 and S2). Average absorbance and standard deviation are shown. In rows, the same capital letter indicates no significant difference according to Games–Howell post hoc test (*p* < 0.05).

	S1M5C01-L	S1M5C01-S	S1M6C01-L	S1M6C01-S	S2M5C01-L	S2M5C01-S	S2M6C01-L	S2M6C01-S
**Carbohydrates**								
d-Cellobiose	0.28 (0.43) A	0.00 (0.00) A	0.07 (0.08) A	0.02 (0.05) A	0.02 (0.04) A	0.24 (0.18) A	0.11 (0.09) A	0.10 (0.11) A
α-d-lactose	0.02 (0.06) A	0.00 (0.00) A	0.00 (0.00) A	0.00 (0.00) A	0.00 (0.00) A	1.40 (0.65) B	0.00 (0.00) A	0.06 (0.09) A
β-methyl-d-glucoside	0.00 (0.00) A	0.53 (0.87) AB	0.00 (0.00) A	0.00 (0.00) A	0.00 (0.00) A	1.54 (0.22) B	0.08 (0.09) A	1.07 (0.61) AB
d-xylose	0.08 (0.09) AB	0.45 (0.79) AB	0.17 (0.09) AB	0.00 (0.00) A	0.49 (0.35) AB	1.52 (0.49) C	0.35 (0.23) AB	0.87 (0.41) BC
i-erythritol	0.02 (0.05) A	0.12 (0.19) A	0.02 (0.06) A	0.05 (0.07) A	0.47 (0.43) A	0.18 (0.22) A	0.08 (0.06) A	0.58 (0.36) A
d-mannitol	1.62 (0.41) A	1.00 (0.73) A	1.63 (0.62) A	0.77 (0.75) A	1.52 (0.53) A	1.80 (0.22) A	1.03 (0.73) A	2.00 (0.00) A
*N*-acetyl-d-glucosamine	0.60 (0.40) AB	0.09 (0.21) A	0.53 (0.11) AB	0.30 (0.16) AB	0.78 (0.42) AB	1.96 (0.06) C	0.38 (0.11) AB	1.30 (0.61) BC
**Carboxylic acids**								
d-glucosaminic acid	0.71 (0.53) AB	0.21 (0.39) A	0.46 (0.45) AB	0.84 (0.72) AB	1.38 (0.49) B	0.99 (0.35) AB	0.72 (0.53) AB	1.17 (0.47) BC
d-galactonic acid γ-lactone	0.88 (0.04) B	0.73 (0.55) AB	0.63 (0.37) AB	0.12 (0.17) A	1.06 (0.20) BC	1.10 (0.23) BC	0.59 (0.24) AB	1.53 (0.28) C
d-galacturonic acid	1.80 (0.05) C	0.49 (0.52) A	0.61 (0.46) A	0.63 (0.79) AB	1.46 (0.59) AB	1.35 (0.14) AB	1.54 (0.51) AB	1.60 (0.09) B
γ-hydroxybutyric acid	0.00 (0.00) A	0.12 (0.18) A	0.04 (0.07) A	0.06 (0.09) A	0.12 (0.10) A	0.10 (0.12) A	0.04 (0.07) A	0.07 (0.07) A
Itaconic acid	0.00 (0.00) A	0.10 (0.18) A	0.10 (0.24) A	0.32 (0.35) A	1.05 (0.57) AB	1.43 (0.43) B	0.23 (0.44) A	1.59 (0.36) B
α-ketobutyric acid	0.00 (0.00) A	0.00 (0.00) A	0.05 (0.13) A	0.00 (0.00) A	0.00 (0.00) A	0.00 (0.00) A	0.00 (0.00) A	0.00 (0.00) A
d-malic acid	0.00 (0.00) A	0.03 (0.08) A	0.11 (0.09) A	0.00 (0.00) A	0.21 (0.23) A	0.43 (0.43) A	0.06 (0.10) A	0.39 (0.21) A
**Phosphorylated chemicals**								
Glucose-1-phosphate	0.05 (0.08) A	0.00 (0.00) A	0.03 (0.07) A	0.00 (0.00) A	0.20 (0.12) AB	0.81 (0.18) C	0.10 (0.11) AB	0.34 (0.17) B
d.l-α-glycerol phosphate	0.37 (0.06) AB	0.15 (0.20) A	0.24 (0.06) AB	0.15 (0.14) A	0.41 (0.08) B	0.41 (0.15) B	0.29 (0.12) AB	0.30 (0.05) AB
Aromatic compounds								
2-hydroxybenzoic acid	0.00 (0.00) A	0.00 (0.00) A	0.00 (0.00) A	0.00 (0.00) A	0.00 (0.00) A	0.00 (0.00) A	0.00 (0.00) A	0.00 (0.00) A
4-hydroxybenzoic acid	0.86 (0.77) BC	0.00 (0.00) A	0.54 (0.60) AB	0.00 (0.00) A	0.00 (0.00) A	1.43 (0.31) C	0.38 (0.42) AB	1.12 (0.39) BC
**Esters**								
Pyruvic acid methyl ester	0.79 (0.07) AB	0.62 (0.61) AB	0.47 (0.20) A	0.40 (0.47) A	0.94 (0.37) BC	1.04 (0.20) BC	0.49 (0.32) AB	1.35 (0.21) C
Amino acids								
l-arginine	0.82 (0.38) B	0.38 (0.48) AB	1.03 (0.79) B	0.09 (0.17) A	1.34 (0.73) B	1.16 (0.57) B	0.49 (0.38) AB	1.44 (0.62) B
l-asparagine	1.74 (0.29) B	1.03 (0.81) AB	1.40 (0.63) AB	0.51 (0.73) A	1.39 (0.68) AB	2.00 (0.00) B	1.29 (0.63) AB	1.94 (0.07) B
l-phenylalanine	0.16 (0.09) A	0.03 (0.06) A	0.07 (0.08) A	0.00 (0.00) A	0.17 (0.16) A	0.15 (0.13) A	0.11 (0.09) A	0.10 (0.08) A
l-serine	1.61 (0.13) A	1.36 (0.63) A	1.98 (0.04) A	0.95 (0.90) A	1.53 (0.47) A	1.61 (0.27) A	1.42 (0.58) A	2.00 (0.00) A
l-threonine	0.22 (0.07) A	0.11 (0.13) A	0.02 (0.04) A	0.03 (0.08) A	0.32 (0.26) A	0.16 (0.14) A	0.11 (0.14) A	0.12 (0.09) A
Glycyl-l-Glutamic acid	0.27 (0.08) A	0.22 (0.36) A	0.67 (0.27) AB	0.24 (0.41) A	0.88 (0.47) B	0.26 (0.22) A	0.25 (0.16) A	0.45 (0.16) AB
**Amines**								
Phenylethylamine	0.00 (0.00) A	0.00 (0.00) A	0.00 (0.00) A	0.00 (0.00) A	0.65 (0.23) B	1.58 (0.27) C	0.00 (0.00) A	1.79 (0.26) C
Putrescine	0.70 (0.24) A	0.19 (0.30) A	1.01 (0.22) B	0.38 (0.47) A	0.53 (0.46) A	0.54 (0.39) A	0.73 (0.40) AB	1.30 (0.09) B
**Polymers**								
Tween 40	1.14 (0.45) B	1.01 (0.48) B	0.96 (0.63) B	0.35 (0.38) A	0.94 (0.42) B	1.48 (0.16) B	0.58 (0.32) A	1.17 (0.22) B
Tween 80	1.00 (0.35) A	0.59 (0.62) A	1.14 (0.62) A	0.39 (0.43) A	0.88 (0.31) A	0.72 (0.22) A	0.88 (0.52) A	1.08 (0.33) A
α-cyclodextrin	0.02 (0.04) A	0.00 (0.00) A	0.06 (0.07) A	0.02 (0.05) A	0.04 (0.10) A	0.18 (0.20) A	0.05 (0.08) A	0.02 (0.04) A
Glycogen	0.06 (0.09) A	0.41 (0.43) AB	0.12 (0.07) A	0.04 (0.06) A	0.05 (0.13) A	0.84 (0.23) B	0.12 (0.10) A	1.00 (0.36) B

## Appendix A

**Table A1:** Experimental uncertainty and precision of the measurements of absorbance used for analyzing the carbon-source utilization patterns of the microbial communities associated with lichen thalli and substrates. Test samples were randomly chosen from each sample batch at each timepoint.

		Thalli						Substrates					
		48 h (S2M4C03-L) ^1^			72 h (S1M5C14-L)			48 h (S2M5C03-S)			72 h (S2M1C03-S)		
Well	Carbon-Source	RSD ^2^	Mean	CI ^3^	RSD	Mean	CI	RSD	Mean	CI	RSD	Mean	CI
1	Water (blank)	0.76	0.577	0.011	6.31	0.408	0.064	13.79	0.505	0.173	8.59	0.198	0.042
2	Pyruvic acid methyl ester	2.72	1.054	0.071	2.31	1.147	0.066	1.20	1.523	0.045	3.26	1.402	0.114
3	Tween 40	1.85	1.261	0.058	23.41	1.520	0.884	5.58	2.011	0.279	5.24	1.604	0.209
4	Tween 80	4.73	0.963	0.113	11.93	1.983	0.588	2.53	1.639	0.103	5.27	1.161	0.152
5	α-cyclodextrin	0.49	0.627	0.008	6.53	0.399	0.065	3.81	0.512	0.048	7.27	0.436	0.079
6	Glycogen	2.85	0.614	0.044	2.30	0.437	0.025	11.54	1.294	0.371	3.42	1.039	0.088
7	d-cellobiose	0.83	0.625	0.013	7.88	0.414	0.081	8.08	0.962	0.193	4.69	0.530	0.062
8	α-d-lactose	2.20	0.618	0.034	3.08	0.406	0.031	2.33	1.597	0.093	6.08	1.770	0.267
9	β-methyl-d-glucoside	2.16	0.604	0.032	7.04	0.408	0.071	3.25	2.024	0.164	1.33	1.977	0.065
10	d-xylose	1.85	1.572	0.072	11.23	0.706	0.197	1.47	1.677	0.061	5.90	1.414	0.207
11	i-erythritol	3.58	0.622	0.055	9.91	0.460	0.113	6.27	0.774	0.121	10.32	0.473	0.121
12	d-mannitol	1.37	1.473	0.050	2.92	3.016	0.219	1.30	2.383	0.077	5.18	2.045	0.263
13	*N*-acetyl-d-glucosamine	1.18	0.634	0.019	6.41	1.140	0.182	6.51	2.345	0.379	5.43	2.332	0.314
14	d-glucosaminic acid	0.87	1.367	0.030	11.71	0.594	0.173	1.02	1.523	0.039	4.63	1.831	0.210
15	Glucose-1-phosphate	2.70	0.715	0.048	7.63	0.447	0.085	11.65	1.530	0.443	2.06	1.119	0.057
16	d.l-α-glycerol phosphate	1.01	0.897	0.023	5.73	0.635	0.090	10.21	1.140	0.289	2.76	0.494	0.034
17	d-galactonic acid-gamma-lactone	3.39	1.723	0.145	7.88	1.465	0.287	1.96	1.581	0.077	5.82	1.860	0.269
18	d-galacturonic acid	2.92	2.280	0.166	7.70	0.807	0.154	11.80	2.135	0.626	5.01	2.245	0.279
19	2-Hydroxy benzoic acid	5.42	0.167	0.023	5.73	0.107	0.015	1.17	1.702	0.049	2.57	0.243	0.016
20	4-Hydroxy benzoic acid	1.52	0.497	0.019	3.57	1.788	0.159	3.59	1.559	0.139	5.04	1.588	0.199
21	γ-hydroxybutyric acid	6.12	0.758	0.115	6.60	0.477	0.078	16.53	0.680	0.279	16.72	0.352	0.146
22	Itaconic acid	4.39	0.986	0.107	2.35	0.419	0.024	3.65	1.780	0.161	2.71	2.311	0.156
23	α-ketobutyric acid	6.81	0.250	0.042	9.45	0.132	0.031	1.97	0.456	0.022	9.67	0.216	0.052
24	d-malic acid	1.65	0.708	0.029	6.07	0.564	0.085	8.97	0.513	0.114	4.07	0.877	0.089
25	l-arginine	6.76	0.864	0.145	6.31	2.071	0.325	5.84	2.145	0.311	1.36	2.649	0.090
26	l-asparagine	1.44	1.804	0.065	1.62	3.016	0.121	2.32	2.307	0.133	2.33	3.011	0.174
27	l-phenylalanine	3.04	0.639	0.048	11.42	0.214	0.061	12.50	0.484	0.150	11.89	0.318	0.094
28	l-serine	8.25	1.028	0.211	1.29	2.730	0.088	3.55	2.553	0.225	14.38	2.384	0.851
29	l-threonine	7.02	0.783	0.137	14.97	0.463	0.172	15.38	0.596	0.228	6.31	0.266	0.042
30	Glycyl-l-glutamic acid	1.44	1.096	0.039	1.45	1.448	0.052	8.15	0.996	0.202	12.22	0.701	0.213
31	Phenylethylamine	9.75	0.578	0.140	18.82	0.279	0.130	7.06	0.583	0.102	4.34	1.623	0.175
32	Putrescin	2.43	0.656	0.040	3.23	1.655	0.133	4.16	1.496	0.154	7.53	1.175	0.220

^1^ The nomenclature for each composite sample indicates the site (S1 or S2), then the mycobiont (M1, M4 or M5), then the cyanobiont (C03 or C14) and whether it originates from the lichen thallus (-L) or from the substrate (-S). ^2^ RSD: relative standard deviation. ^3^ CI: confidence interval.
